# Nearshore Depth Estimation Using Fine-Resolution Remote Sensing of Ocean Surface Waves

**DOI:** 10.3390/s23239316

**Published:** 2023-11-21

**Authors:** Mengyuan Liu, Shouxian Zhu, Shanling Cheng, Wenjing Zhang, Guangsong Cao

**Affiliations:** 1 College of Oceanography, Hohai University, Nanjing 210098, China; 191311040008@hhu.edu.cn (M.L.); chengshanling@gsafety.com (S.C.); 2 College of Meteorology and Oceanography, National University of Defense Technology, Changsha 410073, China; zhangwenjing21@nudt.edu.cn; 3 Institute of Water Science and Technology, Hohai University, Nanjing 210098, China; 20210507@hhu.edu.cn

**Keywords:** bathymetry, remote sensing, ocean surface wave, fast Fourier transform, spatial profile measurement

## Abstract

In the field of water depth inversion using imagery, the commonly used methods are based on water reflectance and wave extraction. Among these methods, the Optical Bathymetry Method (OBM) is significantly influenced by bottom sediment and climate, while the wave method requires a specific study area. This study introduces a method combining the FFT and spatial profile measurement to invert the wavelength of the wave bathymetry method (WBM), which enhances accuracy and reduces workload. The method was applied to remote sensing images of Sanya Bay in China, obtained from the Worldview satellite. The average error of the inverted depth results after applying the wavelength inversion technique was 15.9%, demonstrating consistency with the depth measurements obtained through the OBM in clear water of the bay. The WBM has notable advantages over the OBM, as it is unaffected by water quality. In addition, the influence of wave period on the accuracy of water depth retrieval was theoretically evaluated, revealing that a larger wave period leads to a better depth measurement. The depth measurement from two images with different wave periods aligned with the theoretical analysis. These results showcase the applicability and potential of the WBM for accurately estimating water depth in various coastal environments.

## 1. Introduction

Coastal bathymetric information is essential for a wide range of activities, including marine engineering projects, maritime safety, fisheries, tourism, military operations, and marine scientific research. Traditional depth measurement methods, such as shipborne echo sounders or sonar, are known for their high accuracy. However, these methods are time-consuming and labor-intensive, and their application is limited by coastal navigation restrictions. To overcome these issues, a new depth measurement method has been developed using satellite remote sensing technology, which offers advantages such as speed, wide coverage, synchronous acquisition, and high resolution [1,2,3,4,5,6,7,8].

Methods for water depth measurement using remote sensing can be broadly classified into the synthetic aperture radar (SAR) method based on hydrodynamic modulation and the visible spectral remote sensing method based on water body optical parameters. The latter can be further divided into several categories: the Optical Bathymetry Method (OBM) based on the underwater reflected visible light [9,10], and depth measurement methods based on remote sensing wave information extraction [11,12]. Among all these methods, the Optical Bathymetry Method (OBM) is the most widely used [13,14,15,16]. The OBM is based on the principle that the total reflected energy of electromagnetic waves from a water column (including the water surface, water body, and seabed) varies with water depth [17,18,19,20,21,22,23,24]. However, the OBM requires specific criteria for the seabed sediment and climate conditions of the study area. Additionally, accurately measuring water depth in areas with high turbidity and rough sea conditions poses significant challenges for the OBM.

In wave-dominated nearshore areas, water depth can be obtained using the physical relationship between wave speed and depth. The Radon-Augmented Sentinel-2 satellite imagery to derive wave patterns and the regional bathymetry method utilize the measurement of wave patterns from optical remote sensing images, which exhibit dark and bright stripes on ocean waves. The wavelength can be derived by measuring these stripes. The motion of the stripes estimated from two or more images taken within a short time interval can be used to calculate wave speed [25,26]. Wavelength and wave speed mainly depend on the water depth in shallow water, where the wave frequency is almost stable within a limited area. Therefore, remote sensing wave inversion can be used to estimate water depth. Photography [27], video [28,29], and marine radar [30,31,32,33,34,35] have also been used to obtain wave patterns for bathymetry estimation.

Early methods for extracting wavelength were mainly applied in optical remote sensing imaging. Leu extracted wavelength from SPOT-3 panchromatic images using the Fast Fourier Transform (FFT) method to obtain the inverted water depth in Taichung Harbor [36]. This method does not rely on other environmental data, such as water quality and bottom reflectance, and does not require additional observed water depths in bathymetric operations [36,37,38,39]. It has practical application value as it does not depend on the simultaneously observed wind speed and surface flow velocity in radar image analysis. Poupardin et al. inverted the wavelength and water depth in the Saint-Pierre coastal area using SPOT-5 images based on the wavelet transform [40]. Li et al. used a single image to estimate wavelength and water depth in Sanya Bay, China, mainly through spatial profile measurement (SPM) [41]. Other satellites also provide fine-resolution optical images for wave and bathymetry derivation [42,43,44,45,46,47,48,49].

Existing applications of the wave bathymetry method through optical remote sensing (WBM-ORS) wave depth measurement method have shown that both the Fast Fourier Transform (FFT) and SPM methods yield accurate depth measurement through wavelength inversion [40,50]. However, the FFT method still has limitations in certain scenarios [51,52]. Shen et al. conducted simulation research and found that the FFT method requires a specific image resolution and subimage size, with the subimage length being 4–8 times the wavelength [53]. Furthermore, the FFT method is unable to accurately extract wavelengths at the image edges. In areas with large water depth fluctuations and at the image edges, the FFT method struggles to provide precise wavelength extraction due to the requirement for subdomains of several wavelengths to compensate the randomness of surface waves [36,54].

To address this issue, we introduced the SPM method as a supplement to the FFT method for areas where the extraction results are not satisfactory. While the SPM method requires a large amount of manual operation and heavy workload, combining the SPM and FFT methods for depth measurement can improve the accuracy of measurement and reduce the workload. In addition, we have also investigated the influence of wave period on the FFT method, which helps in selecting suitable images for inversion. Overall, by combining the strengths of both the FFT and SPM methods and considering the influence of wave period, we believe that our approach can achieve more accurate and efficient depth measurements using the WBM-ORS.

This paper mainly focuses on the depth inversion method based on the combination of FFT and SPM. The novelty of this paper lies in the combination of FFT and SPM methods for depth inversion and comparison with the traditional OBM, as well as the analysis of wave periods in the images. The structure of this paper is as follows: Section 1 introduces the history of depth inversion and presents the research methodology of this paper. Section 2 describes the study area and research methods. Section 3 uses the combined FFT and SPM method to extract depth and compares it with the OBM. Section 4 analyzes the influence of wave period on depth inversion.

## 2. Study Area and Methods

### 2.1. Study Area

**Figure 1:**
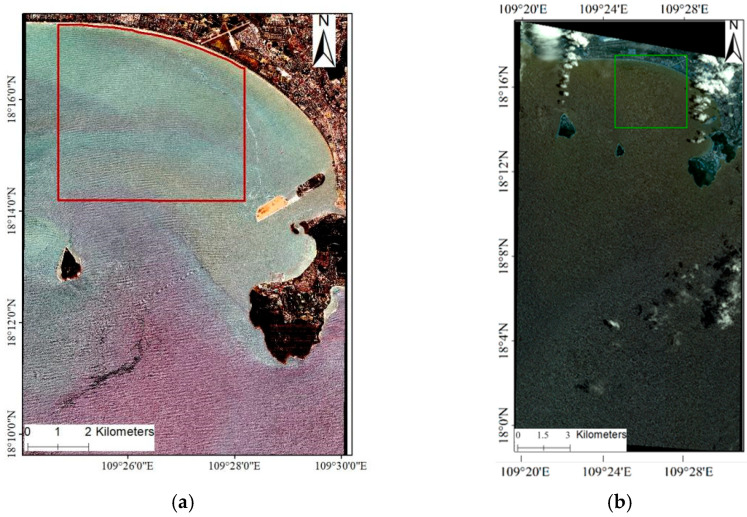
Geographical location of Sanya Bay. (**a**) The Worldview-2 covering Sanya Bay on 5 August 2019. (The red color polygon represents the study area). (**b**) The Quickbird image covering Sanya Bay on 30 June 2009. (The green color polygon represents the study area).

We conducted water depth inversion using the WBM-ORS in Sanya Bay, located in the southern Hainan island of China (shown in Figure 1). Sanya Bay is a popular tourist destination known for its tropical marine climate, stunning white-sand beaches, and pristine water quality. Ensuring the safety of swimmers and accurately predicting the marine environment in the bay require frequent bathymetry measurements.

### 2.2. Study Data

In this study, we utilized an optical remote sensing image captured by the Worldview satellite to perform water depth inversion in Sanya Bay. The image was acquired at UTC 3:30 on 5 August 2019. It consisted of a panchromatic band with a resolution of 0.5 m and multi-spectral bands (including red, green, blue, and near-infrared bands) with a resolution of 2 m. However, due to significant noise in the panchromatic data, we solely relied on the multi-spectral data for our analysis.

Figure 1 shows the muti-spectral image, clearly depicting the wavy characteristics of the bay. For bathymetry inversion, we selected a nearshore area within the red frame of Figure 1. This area was chosen based on the wave-shaped information derived from remote sensing and was ensured to be free from isolated small islands or artificial structures.

**Figure 2:**
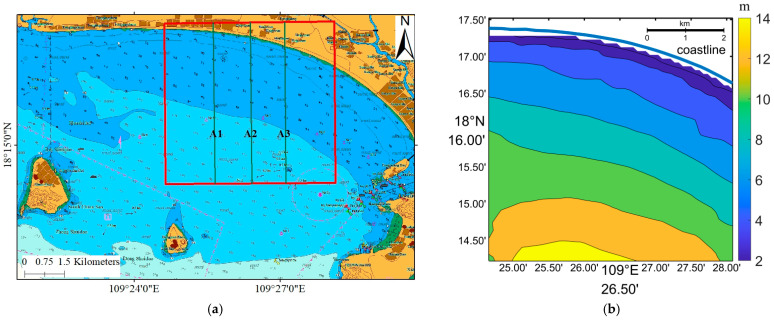
(**a**) The nautical chart of Sanya Bay and (**b**) the water depth contour within the red frame of the nautical chart. The red square is study area, and blue line represents the shoreline.

To assess the accuracy of our bathymetry results, we compared them with the nautical chart from China Navigation Publications published on 7 September 2020, featuring a scale of 1:25,000 (Figure 2a). The original water depth data used to generate the nautical chart were obtained in 2018, according to the source diagram on the chart. Figure 2b illustrates the water depth contours generated based on the water depths provided in the nautical chart. Within the red frame, there were a total of 207 points with observed water depth data, although only a portion of the data is shown in the figure.

To compare our method with the traditional approach, we also utilized some Landsat-8 data for spectroscopy verification, which have a resolution of 30 m. The details of the datasets are described in Table 1. Additionally, a Quickbird image captured at UTC 3:35 on 30 June 2009 was also used to invert water depth using the WBM-ORS in Sanya Bay. The resolutions for the panchromatic band and multi-spectral band of the image are 0.6 m and 2.4 m, respectively. The QuickBird image covered the Sanya Bay region and provided valuable surface wave information for our study. Neither of the images displays the presence of passing ships.

In this study, we utilized the ArcGIS 10.5 software for data processing. ArcGIS is a desktop application geographic information system platform (https://www.arcgis.com (8 June 2021)) that offers a wide range of functionalities, including spatial data visualization, editing, querying, retrieval, statistical analysis, report generation, spatial analysis, and advanced cartography. To account for the influence of solar radiation and atmospheric reflection on the reflectance of the sea surface, we performed radiometric and atmospheric corrections on the images using ArcGIS. Additionally, positional errors can exist in the images, leading to differences in the coordinates of the nautical chart and remote sensing images. To ensure the reliability of our results, we conducted geometric corrections and coordinate transformations on the images using ArcGIS. In addition, to reduce the influence of land on bathymetry inversion, we separated the images using ArcGIS. These corrections enhanced the accuracy and reliability of the data, as well as the precision of the bathymetry results.

The reanalysis wave products from European Center for Medium Weather Forecasting (ECMWF, https://cds.climate.copernicus.eu/cdsapp#!/dataset/reanalysis-era5-single-levels?tab=form (20 July 2021)) were used to provide wave period in deep water ($T_{d}$), with a spatial resolution of 0.125° × 0.125°. The ECMWF is an internationally recognized weather forecasting research organization consisting of 34 countries, including Austria, France, and Germany. Their data coverage includes the global sea. The reanalysis data from ECMWF include the wind field at 10 m above the sea surface, as well as wave data. The spatial resolution of the wave data varies and includes 0. 75° × 0.75°, 0.50° × 0.50°, 0. 25° × 0.25°, 0.125° × 0.125°.

### 2.3. WBM-ORS 

In this study, we utilized WBM-ORS to estimate water depth from remote sensing data. Firstly, we extracted wavelength information of ocean surface waves from optical images using Fast Fourier Transform (FFT) and SPM methods. Then, we employed the wave dispersion relationship to infer water depth using wavelength information of ocean waves.

Discrete Fourier Transform (DFT) is a common method to extract wavelength. Using the DFT method, the remote sensing image can be divided into a number of N×N pixel sub-images, the resolution of an image is marked as ΔX, and the pixel index at a point is $\left(m_{1},m_{2}\right)$. The Fourier coefficient and two-dimensional wavenumber spectrum are [36]

(1) $$F(k_{x},k_{y})=\frac{1}{N^{2}}\sum_{m_{2}=0}^{N-1}\left[\sum_{m_{1}=0}^{N-1}X(m_{1},m_{2})\cdot e^{-in_{x}k_{0}m_{1}\Delta X}\right]\cdot e^{-in_{y}k_{0}m_{1}\Delta X}$$

(2) $$\psi (k_{x},k_{y})={\left|F(k_{x},k_{y})\right|}^{2}$$

where D=NΔX is the length of sub-image; the values of $n_{x}$ and $n_{y}$ are 1, 2, 3, …, N; $k_{x}=n_{x}k_{0}$ and $k_{y}=n_{y}k_{0}$ are wavenumbers in the x and y directions, respectively; $k_{0}=2\pi /D$ is the wavenumber resolution. The ranges of wavenumbers in the x and y directions in the FFT method are both

(3) $$\left[-\frac{N}{2}k_{0},\frac{N}{2}k_{0}\right]$$

In a two-dimensional spectrogram, the spectral peak located at $\left(k_{px},k_{py}\right)$ represents the characteristic of a dominant wave system; thus, the inverted wavelength and wave direction from wavenumber are

(4) $$L=\frac{2\pi }{\sqrt{{\left(k_{px}\right)}^{2}+{\left(k_{py}\right)}^{2}}}$$

(5) $$\theta_{p}=\arctan\left(\frac{k_{py}}{k_{px}}\right)$$

In this study, the FFT method was used in wavelength inversion from remote sensing images.

The dispersion relationship of sea waves can be approximatively given using Linear Airy Wave theory [46]:

(6) $$\omega^{2}=gk\tan hkh$$

where ω=2π/T is the radian frequency, *T* is the wave period, *g* is the acceleration due to gravity, k=2π/L is the wavenumber, *L* is the wavelength, h is the water depth. In deep water of *h* > *L*/2, tanh⁡kh is very close to 1, and the dispersion relationship can be simplified as

(7) $$T=\sqrt{\frac{2\pi L}{g}}$$

The wavelength is nearly independent of water depth in deep water, where the remote sensing wavelength cannot be used in bathymetry. In shallow water (h<L/2), the transformation of Equation (6) expresses the relationship between the water depth and wavelength:

(8) $$h=\frac{L}{2\pi }\mathrm{arctanh}\left(\frac{2\pi L}{gT^{2}}\right)$$

Through the use of Equation (8), the water depth can be inverted by measuring the shallow-water wavelength.

### 2.4. Wave Period

Generally, the wave period is conserved as the wave moves from deep water toward shallow water, and the wave period in shallow water ($T_{s}$) approximately equals that in adjacent deep water ($T_{d}$). Using Equation (7), Leu and Chang [36] and Li et al. [41] both estimated $T_{d}$ using the remote sensing wavelength in deep water. In this study, the Worldview image shown in Figure 1 did not cover a wide area of deep water, making it challenging to estimate the wave period $T_{d}$ directly from the remote sensing wavelength. To overcome this limitation, we utilized reanalysis wave products from ECMWF to provide $T_{d}$.

**Figure 3:**
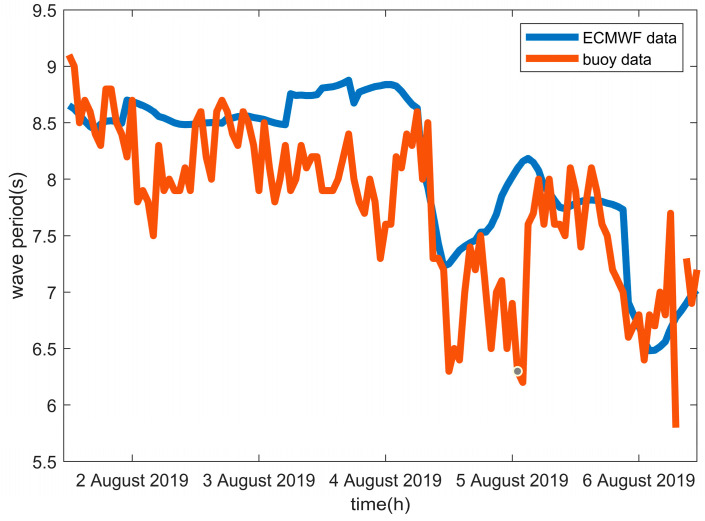
Wave period comparison between ECWMF data and buoy data in the South China Sea.

To validate the accuracy of the ECMWF data, we utilized hydro-meteorological buoy data from the China Marine Monitoring Center. The buoy data, with a temporal resolution of 1 h, cover the time period from 2 August 2019 to 6 August 2019, and are located at 115.47° E and 19.87° N. In order to align the buoy data, we compared them with the corresponding ECMWF data at 115.5° E and 20.0° N, also spanning from 2 August 2019 to 6 August 2019, and having a temporal resolution of 1 h. The analysis revealed a strong and significant correlation of 0.70 between the two datasets (Figure 3), confirming the reliability of the ECMWF data.

**Figure 4:**
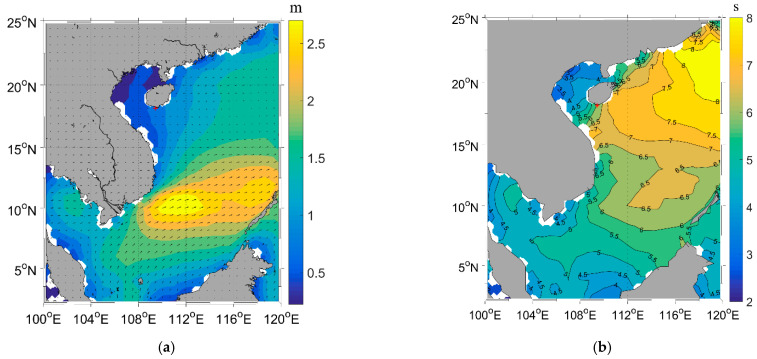
Significant wave height and mean wave period from ECMWF in the South China Sea corresponding to the Worldview image. (**a**) Significant wave height. (**b**) Mean wave period. The red “+” indicates the location of Sanya Bay.

Figure 4 displays the significant wave height and mean wave period from ECMWF in the South China Sea, which corresponds to the areas captured by the Worldview image. The significant wave height ranged from 0.5 to 3 m in the South China Sea and was approximately 1 m near Sanya Bay. Additionally, the mean wave period varied from 2 to 8 s in the South China Sea and was approximately 6 s near Sanya Bay. The inverted wavelength through the FFT method corresponds to the spectral peak; thus, the peak period is required for the bathymetry application of remote sensing wavelength. According to the theoretical analysis of Li [50], the peak period is 1.45 times the mean wave period. Therefore, the peak period corresponding to the Worldview image was estimated to be approximately 8.7 s.

## 3. Results

### 3.1. The Wavelength and Water Depth Inversion from Worldview Data using the FFT Method

In the wavelength inversions using the FFT method, we divided the image into sub-images of different sizes. The sizes of the sub-images were chosen as 32×32, 64×64, 128×128, 256×256, 512×512, and 1024×1024, which corresponded to sub-image lengths of 64, 128, 256, 512, 1024, and 2048 m. The space interval between two adjacent center points of the sub-images was set to 64 m, so the neighboring sub-images may have partial overlap, allowing for more robust and accurate inversion results by incorporating information from adjacent sub-images.

In the sub-images analyzed from Worldview data, we obtained 317, 268, 243, 167, and 24 normal values of wavelengths for sub-image lengths of 64, 128, 256, 512, 1024, and 2048 m, respectively. These values were used to derive water depth measurements, which yielded average errors of 55.4%, 52.7%, 38.0%, 26.6%, 17.3%, and 8.6% when compared to the sea chart, respectively. It is important to note that these measurements were adjusted by subtracting the tide level of 1.22 m (obtained from the tide table of Sanya tide gauge station). Considering the validity and accuracy of the inversion results, the selected sub-image length of 1024 m was chosen for the FFT method.

**Figure 5:**
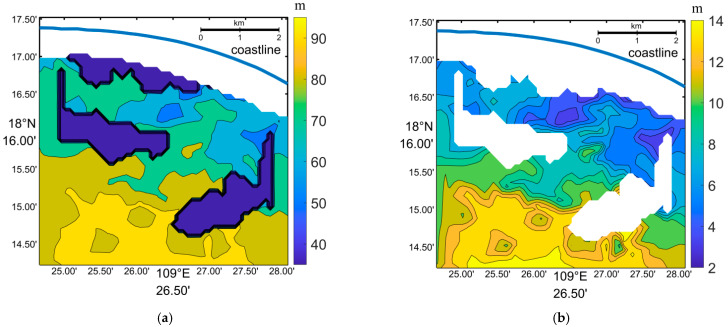
(**a**) Wavelength and (**b**) water depth (tidal level of 1.22 m was subtracted) retrieved from the Worldview image through the FFT method. The blue color polygons represent the area where wavelength is 1024 m, and the blue line represents the shoreline.

Figure 5a displays the inverted wavelengths obtained using this sub-image length. It should be noted that the FFT method failed to invert the wavelength near the water–land boundary, where some points of sub-images were located on the land. The majority of the obtained wavelengths ranged from 53 to 94 m, with some abnormally large values of 1024 m appearing in certain sub-images. Since the wavelength of deep water with a wave period of 8.7 s is approximately 118 m, the abnormally large wavelength of 1024 m cannot be used for bathymetry purposes.

**Figure 6:**
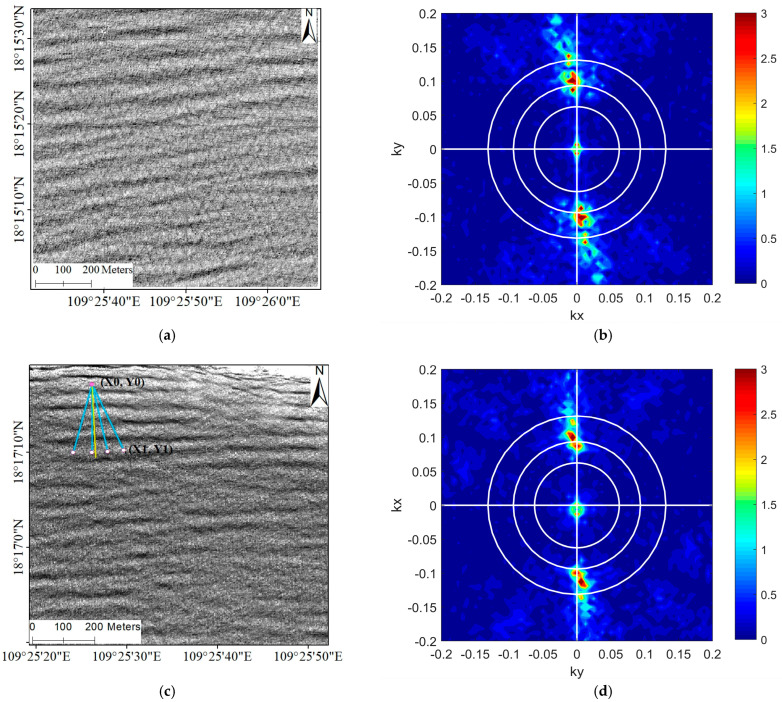
Sub-images and corresponding wavenumber spectrum: (**a**) The sub-image around (109°25′48″, 18°15′17″). (**b**) The wavenumber spectrum calculated by the left sub-image. (**c**) The sub-image around (109°25′35″, 18°16′58″). (**d**) The wavenumber spectrum calculated by the left sub-image.

In Figure 6a, a sub-image centered around (109°25′48″, 18°15′17″) is depicted, demonstrating the clear alignment of wave crests and troughs along the northeast–southwest direction. The wavenumber spectrum, calculated using the FFT method, is shown in Figure 6b. It reveals two symmetrical energy extremes at ($k_{x}$ = 0.0061, $k_{y}$ = 0.0920) and ($k_{x}$ = −0.0061, $k_{y}$ = −0.0920), indicating a dominant wavelength of approximately 68 m. Additionally, there are secondary symmetrical energy extremes at ($k_{x}$ = 0, $k_{y}$ = 0.0061) and ($k_{x}$ = 0, $k_{y}$ = −0.0061). Hence, the wavelength of 1024 m cannot be considered as the dominant wavelength.

Figure 6c presents another sub-image centered around (109°25′35″, 18°16′58″). In this case, the wave crests and troughs are clearly aligned in the north of the area, but there is no continuous pattern in the south. The wavenumber spectrum shown in Figure 6d exhibits two symmetrical energy extremes at ($k_{x}$ = 0, $k_{y}$ = 0.0061) and ($k_{x}$ = 0, $k_{y}$ = −0.0061), along with two secondary symmetrical energy extremes at ($k_{x}$ = 0.0184, $k_{y}$ = −0.0982) and ($k_{x}$ = −0.01840, $k_{y}$ = 0.0982). Therefore, the dominant wavelength determined from this wavenumber spectrum is 1024 m. The other sub-images that resulted in an abnormally large wavelength also had similar characteristics in their wavenumber spectra. In the area depicted in Figure 5, there are a total of 317 water-depth points from the sea chart that were considered for the analysis.

**Figure 7:**
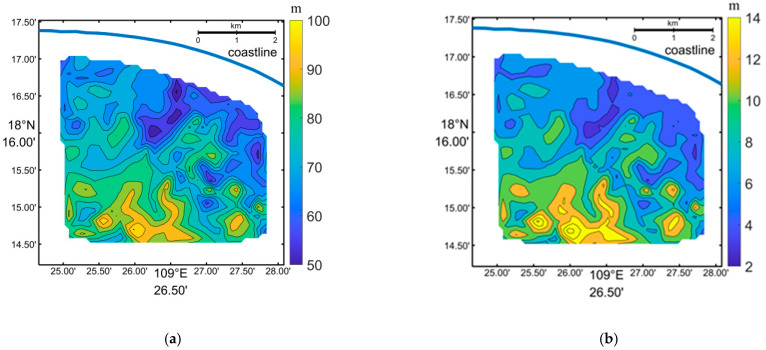
(**a**) Wavelength and (**b**) water depth (tidal level of 1.22 m was subtracted) retrieved through the FFT method from the Worldview image with high-pass-filter preprocessing. The blue line represents the shoreline.

The presence of a spurious peak at near-zero wavenumbers (Figure 6d) indicates the existence of spatial trends or low-frequency spatial variations within the sub-images. To address this issue, a preprocessing step involving de-trending or applying a high-pass digital filter is performed prior to the FFT analysis [55]. Subsequent analysis using FFT reveals a reduction in the presence of abnormally large wavelengths (Figure 7). The corresponding water depth measurements exhibit average errors of 80.9%, 65.3%, 46.9%, 34.7%, 22.7%, and 15.4% compared to the sea chart, when using sub-image lengths of 64, 128, 256, 512, 1024, and 2048 m, respectively. This indicates that an increase in estimation error is the trade-off for expanding the FFT-applicable coverage.

### 3.2. Supplementary Inversions of Wavelength and Water Depth using the SPM Method

For the use of WBM-ORS in Sanya Bay, we also complementally measured the wavelengths using the SPM method near the waterline, and in the sub-images where the FFT method provided abnormal large wavelengths. The SPM method was primarily conducted using the ArcGIS software and involved three steps.

**Figure 8:**
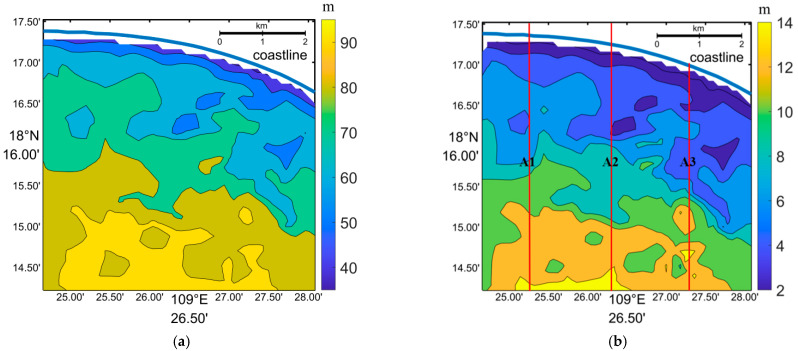
(**a**) Inverted wavelength and (**b**) water depth (subtracting tidal level of 1.22 m) from the Worldview image through WBM-ORS combining FFT and SPM.The blue line represents the shoreline, A1–A3 represent positions of Figure 9.

In the first step, a group of continuous waves with distinguishable crests and troughs were selected. A profile was then extracted across these waves intersecting with the first wave crest at point ($x_{0},y_{0}$), as shown in Figure 8b. Secondly, several points on either side of the profile corresponding to the last wave crest were chosen to form a line. More points ($x_{k},y_{k}$)(*k* = 1, …, *n*) were interpolated along the line. In the third step, the distances between ($x_{0},y_{0}$) and ($x_{k},y_{k}$)(*k* = 1, …, *n*) were calculated. The minimum distance was divided by the number of waves to obtain the average wavelength in the specified area. We utilized the inverted wavelength from the SPM method to fill in the blanks and replace the abnormally large wavelengths in Figure 5a. Both the inverted wavelengths from the FFT and SPM methods are depicted in Figure 8a, and they were used to calculate the corresponding water depths in Figure 8b. Comparing Figure 2b and Figure 8b (inverted water depths), we observe a similar pattern in the bathymetry. The synthetic average bathymetry error was determined to be 15.9%, with the average error through the FFT method being 17.5% and the SPM method inducing an average error of 13.6%.

**Figure 9:**
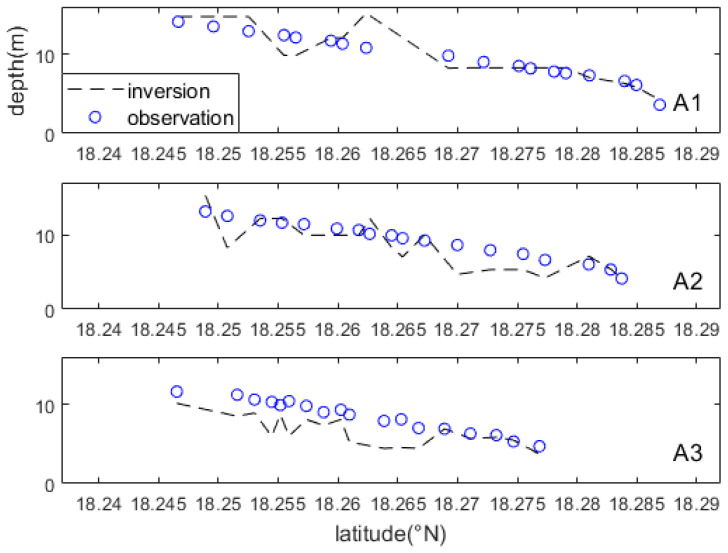
Comparison of the inverted water depths (inversion) through the WBM-ORS and the chart water depths (observation) along three sections. The A1–A3 section positions are shown in Figure 7b.

Figure 9 illustrates the comparison of the inverted water depths and the sea chart depths along sections A1, A2, and A3(shown in Figure 8). The average bathymetry errors for these sections were determined to be 10.5%, 17.8%, and 22.3% respectively. It is evident that the inverted water depths exhibit a similar spatial variation trend as the sea chart depths along these sections. However, along the A3 section, the inverted water depths are consistently lower than the sea chart depths.

**Figure 10:**
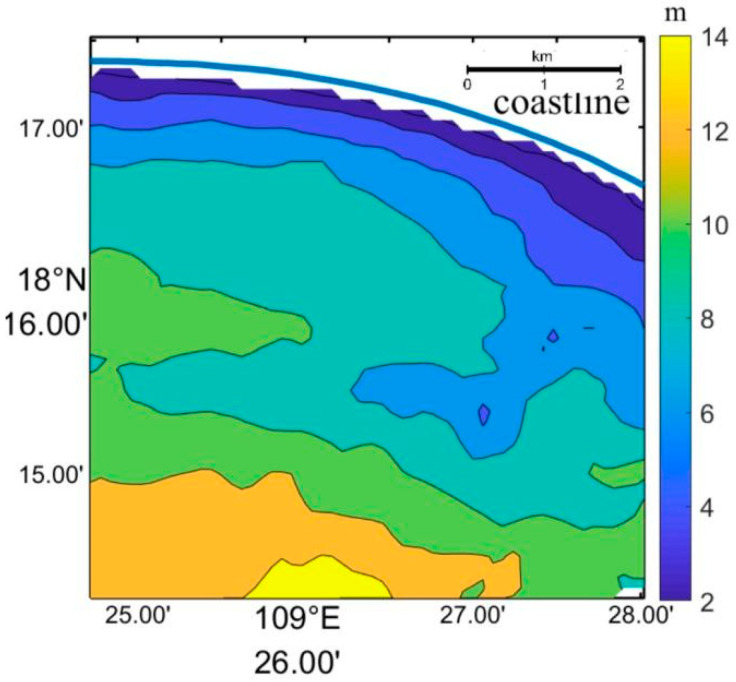
The inverted water depths through the OBM using the Landsat8 image captured on 30 September 2015, with the tidal level of 0.45 m subtracted. The blue line represents the shoreline.

Previous studies [18,19,22] have demonstrated the effectiveness of the OBM for bathymetry in Sanya Bay. In our study, we utilized nine Landsat-8 images with a resolution of 30 m to invert water depths in the bay. Table 1 displays the correlation coefficients between different reflectance (or band ratio) and sea chart depths (NIR denotes the near infrared band). From each image, we selected the reflectance with the largest absolute correlation coefficient to establish the bathymetry model. We employed various forms of equations in the bathymetry models, including linear, logarithmic, power exponent, and exponent forms, respectively:

(9) $$Y=a_{1}X+b_{1}$$

(10) $$Y=a_{2}e^{b_{2}X}$$

(11) $$Y=a_{3}X^{b_{3}}$$

(12) $$Y=a_{4}\ln X+b_{4}$$

where Y is the water depth and X is the selected reflectance or band ratio. $a_{1}$, $b_{1}$, $a_{2}$, $b_{2}$, $a_{3}$, $b_{3}$, $a_{4}$, and $b_{4}$ are the coefficients determined through the least square fitting of reflectance and sea chart depth. Table 2 presents the best bathymetry results for each image, with the tide level subtracted. The average bathymetry errors ranged from 14.8% to 26.3% across these images. With the exception of the image taken on 17 November 2015, the other images yielded an average bathymetry error of less than 20%. Figure 10 presents the inverted water depths using the image captured on 30 September 2015, which has the lowest average error of 14.8%. It is evident that the WBM-ORS can provide bathymetry results almost as accurate as those obtained using the OBM in the clear waters of Sanya Bay.

### 3.3. Assessing Bathymetry Accuracy Affected by Wave Period

**Figure 11:**
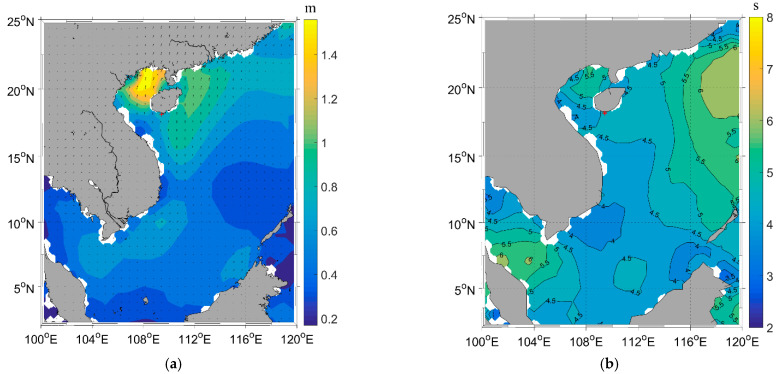
(**a**) Significant wave height and (**b**) mean wave period from ECMWF in the South China Sea corresponding to the Worldview image. The red “+” indicates the location of Sanya Bay.

To evaluate the impact of wave period on bathymetry estimation, we use a Quickbird image captured at UTC 3:35 on 30 June 2009 to invert water depth using the WBM-ORS in Sanya Bay. Figure 11 presents the significant wave height and mean wave period of ECMWF that correspond to the image. In the South China Sea, the significant wave height ranged from 0.1 to 1.5 m, with it being approximately 1 m near Sanya Bay. The mean wave period varied from 2 to 8 s in the South China Sea, and around 4.6 s near Sanya Bay, indicating a peak period of approximately 6.675 s. These wave conditions were considered while evaluating the accuracy of our bathymetry estimation using the QuickBird image.

**Figure 12:**
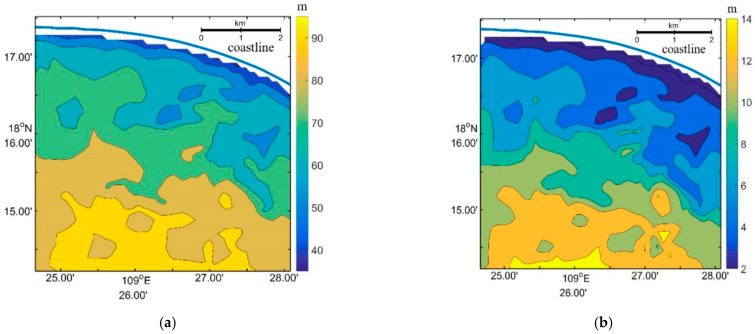
(**a**) Inverted wavelength and (**b**) water depth (subtracting tidal level of 0.62 m) from the Quickbird image by the WBM-ORS combining FFT and SPM. The blue line represents the shoreline.

We applied the FFT method to invert wavelength from the Quickbird image. Similar to the application of the FFT method to the Worldview image described earlier, we used sub-images with different sizes for analysis. The sub-image pixels of the Quickbird image were taken as 32×32, 64×64, 128×128, 256×256, 512×512, and 1024×1024, corresponding to sub-image lengths of 76.8, 153.6, 307.2, 614.4, 1228.8, and 2457.6 m. However, we encountered similar issues with the FFT method near the waterline, as well as abnormal large wavelengths in some sub-images. Among the sub-images around the 317 depth points from the sea chart, only 261, 290, 268, 234, 172, and 32 of them provided normal wavelengths. The water depths inverted using these normal wavelengths exhibited average errors of 57.4%, 41.6%, 31.0%, 25.1%, 24.4%, and 26.8%, respectively, compared to the sea chart. We selected a sub-image length of 1228.8 m for the FFT method. To supplement the wavelength measurements, we employed the SPM method. Figure 11a gives the inverted wavelengths from both the FFT and SPM methods, while the corresponding water depths are shown in Figure 12b. Upon comparing Figure 2b and Figure 11b, the synthetic average bathymetry error is determined to be 22.8%. Additionally, the average error obtained using the SPM method is 19.3%.

Both the Worldview and Quickbird images used in this study contained clear surface-wave information. The Worldview image corresponded to a wave period of 8.7 s, while the Quickbird image corresponded to a wave period of 6.7 s. The average bathymetry error using the Worldview image was 17.5%, and an error of 22.8% was observed when using the Quickbird image.

## 4. Discussions

**Figure 13:**
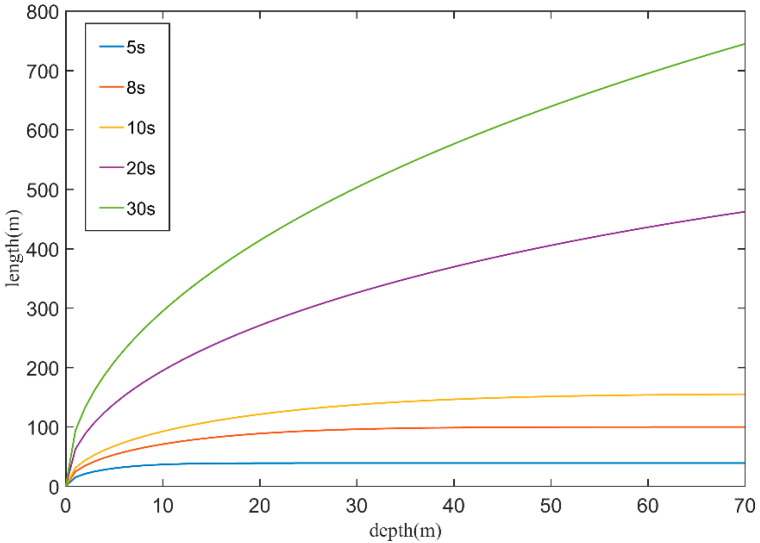
Relationship of wavelength and water depth in shallow water for different wave periods.

To evaluate the impact of wave period on bathymetry error, we calculated the wavelengths for different wave periods (5, 8, 10, 20, and 30 s) and water depths ranging from 0 to 30 m with an interval of 0.1 m by solving Equation (6) numerically. Figure 13 presents the variation in wavelengths with water depth and wave period. Generally, the wavelengths become shorter as waves propagate from deep water to shallow water. In deep water, the wavelengths slowly vary or remain relatively unchanged with changes in water depth. However, in shallow water, the wavelengths exhibit rapid variations with changes in the water depth. Moreover, the change rate of wavelength to water depth is greater for large wave periods compared to smaller ones. This suggests that wave periods can have a significant impact on bathymetry estimation, and their influence may vary with different water depth domains.

To theoretically evaluate the bathymetry depth error impacted by wave period, we developed an error transfer function from the wave dispersion relationship (Equation (6)). We assume that the parameters wave period T and wavelength L are independent variables. Equation (6) becomes

(13) $${\left(\frac{2\pi }{T}\right)}^{2}=\frac{2\pi g}{L}tanh\left(\frac{2\pi h}{L}\right)$$

Differentially expanding both sides of Equation (13), we have

(14) $$-\frac{8\pi^{2}}{T^{3}}\delta T=-\frac{2\pi g}{L^{2}}tanh\left(\frac{2\pi h}{L}\right)\delta L+\frac{2\pi g}{L}{sech}^{2}\left(\frac{2\pi h}{L}\right)\left(\frac{2\pi }{L}\delta h-\frac{2\pi h}{L^{2}}\delta L\right)$$

Then, the bathymetry depth error δh was obtained for small errors δT and δL:

(15) $$\delta h=\left[-\frac{LC}{\pi T}\right]\delta T+\left[\frac{C}{2\pi }+\frac{h}{L}\right]\delta L$$

where $C=sinh\left(\frac{2\pi h}{L}\right)cosh\left(\frac{2\pi h}{L}\right)$. And we have the equation for the stand errors of *h:*

(16) $$\langle {\left(\delta h\right)}^{2}\rangle ={\left[\frac{LC}{\pi T}\right]}^{2}\langle {\left(\delta T\right)}^{2}\rangle +{\left[\frac{C}{2\pi }+\frac{h}{L}\right]}^{2}\langle {\left(\delta L\right)}^{2}\rangle$$

Using Equation (13), we can eliminate variable T and Equation (16) becomes

(17) $$\langle {\left(\delta h\right)}^{2}\rangle =\alpha \langle {\left(\delta T\right)}^{2}\rangle +\beta \langle {\left(\delta L\right)}^{2}\rangle$$

where

$$\alpha =\frac{gL}{2\pi^{3}}{sinh}^{3}\left(\frac{2\pi h}{L}\right)cosh\left(\frac{2\pi h}{L}\right)$$

$$\beta ={\left[\frac{1}{4\pi }sinh\left(\frac{4\pi h}{L}\right)+\frac{h}{L}\right]}^{2}$$

To explain how the stand error of h changes with wavelength L under the condition of a fixed wave period error, we differentiate α with respect to wavelength L.

(18) $$\frac{d\alpha }{dL}=\frac{g}{{2\pi }^{3}}{sinh}^{2}\left(\frac{2\pi h}{L}\right)\left[sinh\left(\frac{2\pi h}{L}\right)cosh\left(\frac{2\pi h}{L}\right)-\frac{6\pi h}{L}{cosh}^{2}\left(\frac{2\pi h}{L}\right)-\frac{2\pi h}{L}{sinh}^{2}\left(\frac{2\pi h}{L}\right)\right]$$

The sign of Equation (18) can be determined by γ, if we assume

(19) $$\gamma =sinh\left(\frac{2\pi h}{L}\right)cosh\left(\frac{2\pi h}{L}\right)-\frac{6\pi h}{L}{cosh}^{2}\left(\frac{2\pi h}{L}\right)-\frac{2\pi h}{L}{sinh}^{2}\left(\frac{2\pi h}{L}\right)$$

The sign of γ is difficult to determine; hence, we have to employ numerical methods for analysis. The steps are as follows:

(1) For a given water depth h, and a series of periods *T_i_* (*i* = 1, 2, 3…), calculate wavelength *L_i_* using the wave dispersion relationship (Equation (6)).
(2) Then, obtain $\gamma_{i}$ using h and *L_i_*.
(3) Repeat step (1) and (2) for different h.

**Figure 14:**
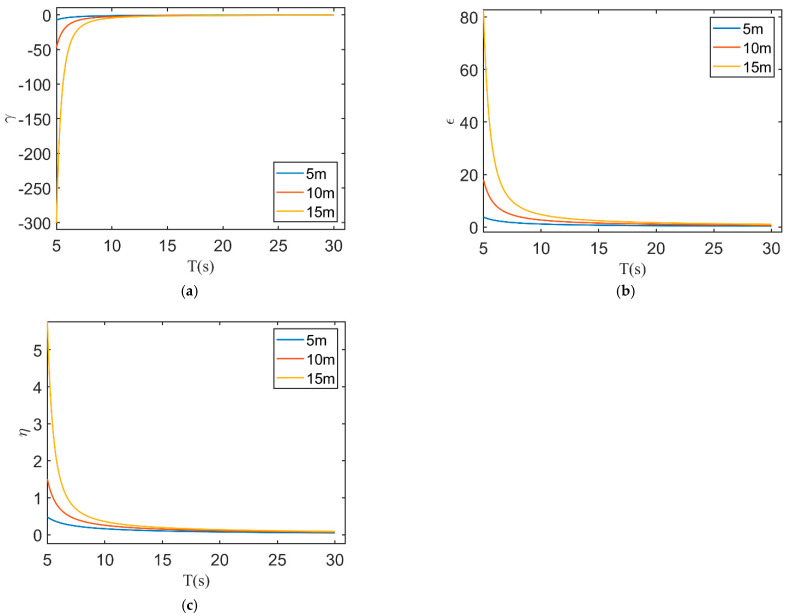
The relationship between (**a**) γ, (**b**) ϵ, and (**c**) η and wave period T for different water depths.

With water depth *h* taken as 5, 10, and 15 m, γ remains less than 0 within the range of wave periods from 5 to 30 s. The absolute value of γ decreases as the period increases, gradually approaching zero (Figure 14a). The cases of wave periods smaller than 5 s and larger than 30 s were also calculated, all resulting in γ less than 0. The absolute value of γ increases as the water depth increases. As the values of γ calculated using Equation (19) are all less than 0, it follows that *d*α*/dL* < 0. For a given water depth *h*, *L* also increases with the increase in wave period T. Given that *d*α*/dL* < 0, α decreases. Consequently, for the same magnitude of period error, a larger *T* leads to a smaller inversion error in water depth.

By defining ϵ=LC/πT in Equation (15), ϵ can also reflect the influence of the period error on the inversion error of water depth. Employing a method similar to the calculation of γ, the variations in ϵ with the period *T* were obtained, as shown in Figure 14b. The inversion error caused by the *T* error decreases as the wave period T increases. The inversion error caused by the *T* error increases with greater water depth.

For β in Equation (17), we have

(20) $$\frac{d\beta }{dL}=-2\frac{h}{L^{2}}\left[\frac{1}{4\pi }sinh\left(\frac{4\pi h}{L}\right)+\frac{h}{L}\right]\left[cosh\left(\frac{4\pi h}{L}\right)+1\right]$$

It is apparent that *dβ*/*dL* < 0. For a given water depth h, when the wave period *T* increases, so does *L*. Due to *dβ*/*dL* < *0*, *β* decreases, thereby resulting in a smaller inversion error in water depth for the same wavelength error when *T* is large. With the definition of η=C/2π+h/L in Equation (15), it can reflect the influence of wavelength inversion error on the inversion error of water depth. As the period *T* increases, η decreases (Figure 14c), indicating that the inversion errors in water depth caused by the wavelength error decrease. The results of our study align with this theoretical evaluation of the bathymetry error impacted by wave period, confirming the consistency between the actual inspection and the theoretical analysis.

Our study area, Sanya Bay, is characterized by abundant surface sediments [56,57]. It is located within the monsoon region, where the dynamic conditions influencing sediment transport, including circulation, waves, and storm surges, exhibit pronounced seasonal variations throughout the year [58,59]. It is important to note that these fluctuations may result in seasonal variations in the underwater topography of Sanya Bay within a given year. Such variations in water depth can also impact the accuracy assessment of remote sensing bathymetry inversion. The South China Sea monsoon exhibits variability at different time scales, including inter-annual and decadal scales [60]. These factors have the potential to influence the accuracy of our nautical charts as representations of ground truth. However, evaluating these impacts requires extensive field observations, which are beyond the scope of this study. We plan to address these aspects in future research endeavors.

## 5. Conclusions

In this study, we developed the WBM-ORS for bathymetry estimation in shallow areas using fine-resolution optical remote sensing images. The method combines the FFT and SPM techniques to invert the wavelength information from the remote sensing data. The FFT method is generally effective but becomes problematic near the waterline. To address this, the SPM method is used as a complementary approach in those problematic sub-images. We applied the WBM-ORS, combining the FFT and SPM, to a Worldview image of Sanya Bay and achieved an average error of 17.5% in water depth inversion.

Furthermore, we investigated the influence of wave period on water depth inversion. Our findings indicated that larger wave periods tend to result in smaller bathymetry errors. The validation using both Worldview and Quickbird images confirmed this, with the Worldview image (wave period of 8.706 s) displaying significantly smaller average bathymetry errors compared to the Quickbird image (wave period of 6.675 s).

Additionally, our study compared the WBM-ORS with the traditional OBM for bathymetry estimation in the clear waters of Sanya Bay. The experiments using nine Landsat-8 images and different functional forms (linear, logarithmic, power exponent, and exponent) resulted in a minimum average bathymetry error of 14.8%. This suggests that the WBM-ORS can provide bathymetry estimates that are nearly as accurate as the OBM, with the added advantage of being insensitive to water quality conditions. This makes the WBM-ORS suitable for application in turbid waters where the OBM might encounter challenges in depth determination.

## Figures and Tables

**Table 1:** Correlation coefficients between water depth and reflectance of Landsate8 images.

Time (Year/Month /Day)	Correlation Coefficients between Water Depth and Reflectance (or Band Ratio)									
	Blue	Green	Red	NIR	Blue/Green	Blue/Red	Blue/NIR	Green/Red	Green/NIR	Red /NIR
27 September 2014	0.18	−0.42	0.63	0.57	0.58	−0.41	−0.4	−0.57	−0.91	−0.93
26 June 2015	0.07	−0.14	0.01	0.01	0.24	0.14	0.1	−0.3	−0.75	−0.7
30 September 2015	−0.23	−0.32	−0.24	−0.25	−0.11	−0.36	−0.33	−0.07	−0.88	−0.88
17 November 2015	−0.35	−0.5	0.21	0.14	−0.33	−0.45	−0.45	0.45	−0.87	−0.9
6 September 2018	−0.64	−0.67	−0.63	−0.63	−0.72	−0.73	−0.72	0.78	−0.55	−0.75
9 July 2020	−0.47	−0.68	−0.49	−0.33	−0.23	−0.49	−0.52	0.05	−0.85	−0.87
2 February 2021	−0.26	−0.47	−0.23	−0.16	−0.07	−0.09	−0.12	0.57	−0.89	−0.86
30 September 2021	−0.65	−0.81	−0.76	−0.72	−0.75	−0.75	−0.76	0.55	−0.84	−0.92
3 December 2021	−0.57	−0.79	−0.76	−0.69	−0.2	−0.49	−0.57	0.03	−0.78	−0.88

**Table 2:** Bathymetry errors using the OBM in Sanya Bay using Landsat8 images.

Time (Year/Month/Day)	Reflectance Ratio (X)	Inversion Formula	Bathymetry Error (%)
27 September 2014	Red/NIR	*Y* = −21.3401 *lnX* + 6.0506	15.8
26 June 2015	Red/NIR	*Y* = −44.9909 *lnX* + 14.5112	18.1
30 September 2015	Green/NIR	*Y* = −26.048 *lnX* + 6.7006	14.8
17 November 2015	Green/Red	*Y* = 10.7541 *X* − 10.8267	26.3
6 September 2018	Green/NIR	*Y* = −21.6731 *lnX* + 9.8358	15.0
9 July 2020	Red/NIR	*Y* = −15.5219 *lnX* + 7.3466	17.0
2 February 2021	Green/NIR	*Y* = −28.4931 *lnX* + 8.0998	19.9
30 September 2021	Red/NIR	*Y* = −11.1087 *lnX* + 4.8556	15.6
3 December 2021	Green/NIR	*Y* = −19.9928 *lnX* + 7.5075	16.3

## Data Availability

Landsat-8 data from publicly available datasets (https://www.gscloud.cn) (18 May 2022).

## References

[1] Cao B., Qiu Z., Zhu S., Tu X. (2016). Shallow Water Bathymetry through Two-Medium Photogrammetry Using High Resolution Satellite Imagery. Acta Geod. Cartogr. Sin..

[2] Huang W.G., Fu B., Zhou C.B., Yang J.S., Li D.L. (2000). Simulation Study on Optimal Currents and Winds for the Spaceborne SAR Mapping of Sea Bottom Topography. Prog. Nat. Sci..

[3] Kaiguo F., Weigen H., Mingxia H., Bin F. (2008). U Progress on Remote Sensing of the Shallow Sea Bottom Topography by SAR. Remote Sens. Technol. Appl..

[4] Zhou G., Li Y., Ren Y., Sheng L., Bai J. (2015). Research of Two-Media Underwater Reefs Depth Measurement Experiment Based on Low-Altitude UAV. Acta Geod. Cartogr. Sin..

[5] Jawak S.D., Vadlamani S.S., Luis A.J. (2015). A Synoptic Review on Deriving Bathymetry Information Using Remote Sensing Technologies: Models, Methods and Comparisons. Adv. Remote Sens..

[6] Luan H.J., He Y.R., Zhu X.L. (2022). Terms of Photogrammetry and Remote Sensing.

[7] Mader D., Richter K., Westfield P., Maas H.-G. (2022). Correction to: Potential of a Non-linear Full-Waveform Stacking Technique in Airborne LiDAR Bathymetry.PFG-Journal of Photogrammetry. Remote Sens. Geoinf. Sci..

[8] Yang F.L., Qi C., Su D.P., Ding S., He Y., Ma Y. (2023). An airborne LiDAR bathymetric waveform decomposition method in very shallow water: A case study around Yuanzhi Island in the South China Sea. Int. J. Appl. Earth Obs. Geoinf..

[9] Niroumand-Jadidi M., Bovolo F., Bruzzone L. (2023). SMART-SDB: Sample-specific multiple band ratio technique for satellite-derived bathymetry. Remote Sens. Environ..

[10] Liu Y.M., Zhao J., Deng R.R., Liang Y., Gao Y., Chen Q., Xiong L., Liu Y., Tang Y., Tang D. (2021). A downscaled bathymetric mapping approach combining multitemporal Landsat-8 and high spatial resolution imagery: Demonstrations from clear to turbid waters. ISPRS J. Photogramm. Remote Sens..

[11] Santos D., Fernández-Fernández S., Abreu T., Silva P.A., Baptista P. (2021). Retrieval of nearshore bathymetry from Sentinel-1 SAR data in high energetic wave coasts: The Portuguese case study. Remote Sens. Appl. Soc. Environ..

[12] Huang L., Fan C., Meng J., Yang J., Zhang J. (2023). Shallow sea topography detection using fully Polarimetric Gaofen-3 SAR data based on swell patterns. Acta Oceanol. Sin..

[13] Zhu W., Ye L., Qiu Z., Luan K., He N., Wei Z., Yang F., Yue Z., Zhao S., Yang F. (2021). Research of the Dual-Band Log-Linear Analysis Model Based on Physics for Bathymetry without In-Situ Depth Data in the South China Sea. Remote Sens..

[14] Liu Y., Tang D., Deng R., Cao B., Chen Q., Zhang R., Qin Y., Zhang S. (2021). An Adaptive Blended Algorithm Approach for Deriving Bathymetry from Multispectral Imagery. IEEE J. Sel. Top. Appl. Earth Obs. Remote Sens..

[15] Zhou G., Su S., Xu J., Tian Z., Cao Q. (2023). Bathymetry Retrieval from Spaceborne Multispectral Subsurface Reflectance. IEEE J. Sel. Top. Appl. Earth Obs. Remote Sens..

[16] Qi J., Zhang D., Ren Z., Cui A., Yin F., Qin J., Zhan J., Zhu J. (2021). Determination of the Initial Value Ranges of Nonlinear Solutions for a Log Ratio Bathymetric Inversion Model and Bathymetry Retrieval. IEEE J. Sel. Top. Appl. Earth Obs. Remote Sens..

[17] Guo X., Qiu Z., Shen W., Luan K. (2017). Shallow Water Depth Inversion in Longwan Port Based on WorldView-2 Remote Sensing Image. J. Mar. Sci..

[18] Shen W., Wang J., Chen M., Hao L., Wu Z. (2023). Research on Bathymetric Inversion Capability of Different Multispectral Remote Sensing Images in Seaports. Sensors.

[19] Shen W., Lih H., Chen M., Xin L. (2023). Evaluation of the bathymetric inversion ability of GF-6 remote sensing images. J. Ocean Technol..

[20] Ma Y., Zhang H., Li X., Wang J., Fan K. (2021). An Exponential Algorithm for Bottom Reflectance Retrieval in Clear Optically Shallow Waters from Multispectral Imagery without Ground Data. Remote Sens..

[21] Zhang G., Zhang W., Zhu S. (2016). Study on the Water Depth Extractionmethod Using Visible Remote Sensing in the Haikou Bay. Mar. Sci. Bull..

[22] Chu M., Zhang H. High resolution multispectral remote sensing for shallow sea topography detection and its application in Lingshui Bay, Hainan. Proceedings of the Second Target Recognition and Artificial Intelligence Summit Forum.

[23] Wang G.-R., Li X.-F., Wang J., Wei Y.-L., Zheng X.-M., Jiang T., Chen X.-X., Wan X.-K., Wang Y. (2022). Development of a Pixel-Wise Forest Transmissivity Model at Frequencies of 19 GHz and 37 GHz for Snow Depth Inversion in Northeast China. Remote Sens..

[24] Yang Q., Chen J., Chen B., Tao B. (2022). Evaluation and Improvement of No-Ground-Truth Dual Band Algorithm for Shallow Water Depth Retrieval: A Case Study of a Coastal Island. Remote Sens..

[25] Salameh E., Frappart F., Almar R., Baptista P., Heygster G., Lubac B., Raucoules D., Almeida L.P., Bergsma E.W.J., Capo S. (2019). Monitoring Beach Topography and Nearshore Bathymetry Using Spaceborne Remote Sensing: A Review. Remote Sens..

[26] Bergsma E.W.J., Almar R., Rolland A., Binet R., Brodie K.L., Bak A.S. (2021). Coastal Morphology from Space: A Showcase of Monitoring the Topography-Bathymetry Continuum. Remote Sens. Environ..

[27] Cox C.S., Munk W.H. (1954). Statistics of The Sea Surface Derived from Sun Glitter. J. Mar. Res..

[28] Stockdon H.F., Holman R.A. (2000). Estimation of Wave Phase Speed and Nearshore Bathymetry from Video Imagery. J. Geophys. Res. Ocean..

[29] Cao B., Deng R., Xu Y., Cao B., Liu Y., Zhu S. (2022). Practical Differences Between Photogrammetric Bathymetry and Physics-Based Bathymetry. IEEE Geosci. Remote Sens. Lett..

[30] Wang Y. (2014). Research Progress of Airborne Laser Bathymetry Technology. J. Geomat..

[31] Guo K., Li Q., Mao Q., Wang C., Zhu J., Liu Y., Xu W., Zhang D., Wu A. (2021). Errors of Airborne Bathymetry LiDAR Detection Caused by Ocean Waves and Dimension-Based Laser Incidence Correction. Remote Sens..

[32] Lowell K., Calder B. (2021). Assessing Marginal Shallow-Water Bathymetric Information Content of Lidar Sounding Attribute Data and Derived Seafloor Geomorphometry. Remote Sens..

[33] Ghosh R., Bovolo F., Bruzzone L., Bovolo F., Pierdicca N. (2022). An FFT-Based CNN-Transformer Encoder for Semantic Segmentation of Radar Sounder Signal. Proceedings of the Image and Signal Processing for Remote Sensing XXVIII.

[34] Vesecky J.F., Stewart R.H. (1982). The observation of ocean surface phenomena using imagery from the SEASAT synthetic aperture radar: An assessment. J. Geophys. Res..

[35] Rieu P., Moreau T., Cadier E., Raynal M., Clerc S., Donlon C., Borde F., Boy F., Maraldi C. (2021). Exploiting the Sentinel-3 Tandem Phase Dataset and Azimuth Oversampling to Better Characterize the Sensitivity of SAR Altimeter Sea Surface Height to Long Ocean Waves. Adv. Space Res..

[36] Leu L.G., Chang H.W. (2005). Remotely Sensing in Detecting the Water Depths and Bed Load of Shallow Waters and Their Changes. Ocean Eng..

[37] Li Y., Zhang X., Cheng L., Xie M., Cao K. (2022). 3D Wave Simulation Based on a Deep Learning Model for Spatiotemporal Prediction. Ocean Eng..

[38] Liu J., Yang Z., Liu Y., Mu C. (2021). Hyperspectral Remote Sensing Images Deep Feature Extraction Based on Mixed Feature and Convolutional Neural Networks. Remote Sens..

[39] Wang Z., Zhao Y., Chen J. (2023). Multi-Scale Fast Fourier Transform Based Attention Network for Remote-Sensing Image Super-Resolution. IEEE J. Sel. Top. Appl. Earth Obs. Remote Sens..

[40] Poupardin A., Idier D., Michele M.D., Raucoules D. (2016). Water Depth Inversion from a Single SPOT-5 Dataset. IEEE Trans. Geosci. Remote Sens..

[41] Li J., Zhang H., Hou P., Fu B., Zheng G. (2016). Mapping the Bathymetry of Shallow Coastal Water Using Single-Frame Fine-Resolution Optical Remote Sensing Imagery. Acta Oceanol. Sin..

[42] Danilo C., Melgani F. (2016). Wave Period and Coastal Bathymetry Using Wave Propagation on Optical Images. IEEE Trans. Geosci. Remote Sens..

[43] Benshila R., Thoumyre G., Najar M.A., Abessolo G., Almar R., Bergsma E., Hugonnard G., Labracherie L., Lavie B., Ragonneau T. (2020). A Deep Learning Approach for Estimation of the Nearshore Bathymetry. J. Coast. Res..

[44] Najar M.A., Benshila R., Bennioui Y., Thoumyre G., Almar R., Bergsma E.W.J., Delvit J.-M., Wilson D.G. (2022). Coastal Bathymetry Estimation from Sentinel-2 Satellite Imagery: Comparing Deep Learning and Physics-Based Approaches. Remote Sens..

[45] Collins A.M., Geheran M.P., Hesser T.J., Bak A.S., Brodie K.L., Farthing M.W. (2021). Development of a Fully Convolutional Neural Network to Derive Surf-Zone Bathymetry from Close-Range Imagery of Waves in Duck, NC. Remote Sens..

[46] Almar R., Bergsma E.W.J., Brodie K.L., Bak A.S., Artigues S., Lemai-Chenevier S., Cesbron G., Delvit J.-M. (2022). Coastal Topo-Bathymetry from a Single-Pass Satellite Video: Insights in Space-Videos for Coastal Monitoring at Duck Beach (NC, USA). Remote Sens..

[47] Gawehn M., Almar R., Bergsma E.W.J., de Vries S., Aarninkhof S. (2022). Depth Inversion from Wave Frequencies in Temporally Augmented Satellite Video. Remote Sens..

[48] Almar R., Bergsma E.W.J., Thoumyre G., Baba M.W., Cesbron G., Daly C., Garlan T., Lifermann A. (2021). Global Satellite-Based Coastal Bathymetry from Waves. Remote Sens..

[49] Abileah R., Gómez-Enri J., Scozzari A., Vignudelli S. (2013). Coherent Ranging with Envisat Radar Altimeter: A New Perspective in Analyzing Altimeter Data Using Doppler Processing. Remote Sens. Environ..

[50] Li R. (2007). On the Relationships of Various Wind Wave Periods. Trans. Oceanol. Limnol..

[51] Ma X., Duan W., Huang L., Qin Y., Yin H. (2022). Phase-Resolved Wave Prediction for Short Crest Wave Fields Using Deep Learning. Ocean Eng..

[52] Zhao D., Xing H., Wang H., Zhang H., Liang X., Li H. (2023). Sea-Surface Small Target Detection Based on Four Features Extracted by FAST Algorithm. J. Mar. Sci. Eng..

[53] Shen S.M., Zhu S.X., Kang Y.Y., Zhang W.J., Cao G.S. (2019). Simulation analysis for remote sensing inversion of wavelength and water depth by the Fast Fourier Transform method. J. East China Norm. Univ. (Nat. Sci.).

[54] Li Y., Wang P., Li Y., Li X. (2014). Evaluation and Comparison of Water Quality of Sanya Bay. Anim. Husb. Feed. Sci..

[55] Bendat J., Piersol A. (2010). Random Data: Analysis and Measurement Procedures: Fourth Edition. Meas. Sci. Technol..

[56] Gao C., Hu M.X., Wu Z.J. (2022). The mechanism of blackening beach in Sanya Bay: The interaction between iron oxides and organic matter. Mar. Geol. Front..

[57] Mao L.J., Zhang Y.Z., Zhang Z.K., Wei L., Ji X.M., Zhu D.K. (2007). Characteristics of sedimentary environments in Sanya Bay of Hainan Island. Mar. Geol. Quat. Geol..

[58] Su J.L. (2004). Overview of the South China Sea circulation and its influence on the coastal physical oceanography outside the Pearl River Estuary. Cont. Shelf Res..

[59] Han L., Ji Q., Jia X., Liu Y., Han G., Lin X. (2022). Significant wave Height Prediction in the South China Sea Based on the ConvLSTM Algorithm. J. Mar. Sci. Eng..

[60] Wang B., Huang F., Wu Z.W., Yang J., Fu X.H., Kikuchi K. (2009). Multi-scale climate variability of the South China Sea monsoon: A review. Dyn. Atmos. Ocean..

